# What shall I do now? State-dependent variations of life-history traits with aging in Wandering Albatrosses

**DOI:** 10.1002/ece3.882

**Published:** 2014-01-23

**Authors:** Deborah Pardo, Christophe Barbraud, Henri Weimerskirch

**Affiliations:** CEBC-CNRS UPR 1934F 79360, Villiers-en-Bois, France

**Keywords:** Bienniality, breeding probability and success, capture–mark–recapture, cost of reproduction, *Diomedea exulans*, failure, survival.

## Abstract

Allocation decisions depend on an organism's condition which can change with age. Two opposite changes in life-history traits are predicted in the presence of senescence: either an increase in breeding performance in late age associated with terminal investment or a decrease due to either life-history trade-offs between current breeding and future survival or decreased efficiency at old age. Age variation in several life-history traits has been detected in a number of species, and demographic performances of individuals in a given year are influenced by their reproductive state the previous year. Few studies have, however, examined state-dependent variation in life-history traits with aging, and they focused mainly on a dichotomy of successful versus failed breeding and non-breeding birds. Using a 50-year dataset on the long-lived quasi-biennial breeding wandering albatross, we investigated variations in life-history traits with aging according to a gradient of states corresponding to potential costs of reproduction the previous year (in ascending order): non-breeding birds staying at sea or present at breeding grounds, breeding birds that failed early, late or were successful. We used multistate models to study survival and decompose reproduction into four components (probabilities of return, breeding, hatching, and fledging), while accounting for imperfect detection. Our results suggest the possible existence of two strategies in the population: strict biennial breeders that exhibited almost no reproductive senescence and quasi-biennial breeders that showed an increased breeding frequency with a strong and moderate senescence on hatching and fledging probabilities, respectively. The patterns observed on survival were contrary to our predictions, suggesting an influence of individual quality rather than trade-offs between reproduction and survival at late ages. This work represents a step further into understanding the evolutionary ecology of senescence and its relationship with costs of reproduction at the population level. It paves the way for individual-based studies that could show the importance of intra-population heterogeneity in those processes.

## Introduction

There is considerable diversity in the patterns of reproductive behavior shown by different species (Stearns 1992). According to the principle of resource allocation (Levins 1968), organisms have to allocate limited energy between two main functions: reproductive and somatic functions. The latter includes growth, maintenance, and energy storage and is inherently linked to survival. In long-lived iteroparous species, individuals are expected to favor their own survival over reproduction as they will have many other occasions to breed (Bell 1980; Reznick 1992). However, allocation decisions can change with an organism's age. Indeed as the residual reproductive value decreases while aging, the investment in reproduction is expected to increase (Pianka and Parker 1975). This prediction might not always be verified as demonstrated by McNamara and Houston (1996) because all individuals of the same age are not necessarily equivalent due to the variations in physiological state and environmental conditions that determine their fitness (VanNoordwijk and DeJong 1986). An organism's fitness can be dependent on its size, quality, or condition associated, for instance, with body reserves, parasite load, foraging skills, immunological state, or territory quality (McNamara and Houston 1996). Therefore, the demographic performances of individuals in a given year may be a function of their current or previous reproductive state (Cam et al. 1998; McElligott et al. 2002; Schaub and von Hirschheydt 2009).

Senescence is defined as an inevitable and irreversible accumulation of damage with age that leads to a loss of function and eventual death (Monaghan et al. 2008). Three theories have been proposed to explain the ultimate causes of the appearance of senescence in wild populations: the mutation accumulation theory (Haldane 1941), the antagonistic pleiotropy theory (Williams 1957), and the disposable soma theory (Kirkwood and Holliday 1979). Detecting senescence in the wild has been challenging due to the necessity of having long-term longitudinal data and because natural selection was believed to eliminate senescent individuals because of their decreased selective value (Monaghan et al. 2008). However, recently, the existence of senescence in the wild has been demonstrated in a number of organisms (e.g., Jones et al. 2008), and two opposite theories on life-history strategies while aging have been described in the literature in the presence of senescence:

1. The terminal investment theory, where old individuals enhance their last forces to some last reproduction events if their condition becomes critical (McNamara et al. 2009; Froy et al. 2013). This theory has received relatively little empirical evidence, some in wild ungulates (Clutton-Brock 1984; Ericsson et al. 2001; Weladji et al. 2010) and in a marsupial (Isaac and Johnson 2005), or in manipulative studies that experimentally increased internal damage in seabirds (Velando et al. 2006) and insects (Morrow et al. 2003).
2. Old individuals might instead reduce inherently their investment in reproduction by diminishing clutch size (Nisbet and Dann 2009) or reducing their breeding frequency (Catry et al. 1998; McElligott et al. 2002), which might help maintaining survival as predicted by the life-history theory (Stearns 1992).

However, despite the observation that age variation in several life-history traits has consistently been detected in a number of species (Jones et al. 2008) and that it has been well established that several life-history traits are state dependent (McNamara and Houston 1996), few studies have examined state-dependent variation in life-history traits with aging which is particularly relevant with respect to senescence (Tavecchia et al. 2005; Descamps et al. 2009; Robinson et al. 2012). As a consequence, little is known about how life-history traits are influenced by breeding states with aging and the different patterns that can emerge between life-history traits. Therefore, using a 50-year dataset of mark–recapture of a long-lived bird, our aim here was to model the age-dependent variations in an array of five key life-history traits simultaneously as a function of the previous breeding state: survival, probability of returning to the breeding grounds, probability of breeding, probability of having an offspring, and probability of bringing the offspring till independence. Previous studies focused mainly on the trade-offs between survival and reproduction while using a simple dichotomy of previous breeding states: breeder or non-breeder the previous year (Tavecchia et al. 2005; Descamps et al. 2009; Robinson et al. 2012). In this study, we intend to go a step further by constructing a gradient of five previous breeding states expected to reflect a gradient of investment in reproduction the previous year: in ascending order, from no reproduction cost to high costs, non-breeder remaining at sea, non-breeder present at breeding grounds, failed breeder during incubation, failed breeder during chick rearing, and successful breeder.

Wandering albatrosses (*Diomedea exulans*) represent good models to investigate this question in a wild population due to their extreme life-history traits, such as low annual reproductive rate, late age at maturity, and long life expectancy. The time scale when aging processes takes place is therefore increased and age-dependent changes more visible. They lay a single egg clutch per breeding event facilitating the assessment of the costs associated with reproduction. Additionally, they are quasi-biennial breeders: due to the length of their breeding cycle (≈1 year), most pairs that successfully raise a chick up to fledging breed every second year (Barbraud and Weimerskirch 2011). Nevertheless, most individuals that fail in their breeding attempt early in the season breed two years in a row, as surprisingly, do a significant proportion ≈ 6% of successful pairs. It is known that body condition is one of the factors determining whether an individual engage into breeding or not at a given time (Weimerskirch 1999) but other factors as age, sex (developed in a sister-paper in Pardo et al. in press), or individual quality can lead to heterogeneities in breeding frequency according to their previous breeding state. In this article, we focus on investigating population level changes in life-history traits according to the previous breeding state and the effect of aging rather than on the individual-level factors or environmental variations causing those changes. This article is structured in two parts. First, we describe how different demographic traits vary according to the previous breeding state. Our first prediction is that individuals attempting to breed in consecutive years (after a failure or a success the previous year) have lower probabilities of survival, return, breeding, hatching, and fledging during the second breeding attempt than birds that did not attempt consecutive-year breeding, due to a higher investment in reproduction and potentially associated costs (Barbraud and Weimerskirch 2011). In a second part, we analyze how incorporating age helps understanding potential changes in the trade-offs between reproduction and survival. Previous studies on wandering albatrosses have demonstrated reproductive (Weimerskirch et al. 2005), survival (Weimerskirch 1992), hormonal (Angelier et al. 2006), and foraging (Angelier et al. 2007; Lecomte 2010) senescence. However, these studies did not explicitly consider the effect of previous breeding state on aging. Additionally, it is suspected that although this species has been long considered as a strictly biennial breeder, some individuals during their lifetime attempt to breed in consecutive years following successful breeding, while other individuals never attempt consecutive-year breeding. In this case, our second prediction is that birds following a strict biennial breeding tactic (i.e., those that take a sabbatical year after a successful breeding attempt) may have a later onset of senescence and/or a lower rate of senescence on both survival and breeding performance than birds attempting consecutive-year breeding (after both a success or a failure) due to higher costs of reproduction in the latter group, possibly related to a more rapid deterioration of their condition (McNamara et al. 2009).

## Material and methods

### Study species and field methodology

The study was conducted at Ile de la Possession, Crozet Islands (46°S; 52°E), southern Indian Ocean. Although the minimal age at first reproduction is five years, on average, individuals first breed at 9–10 years old (Weimerskirch et al. 1997). Birds return to their breeding grounds in November and December, and females lay a single egg in late December – early January. Both parents incubate alternatively until hatching in March. Chicks are reared for ≈ 280 days and fledged in November, when parents progressively reduce chick attendance (Weimerskirch and Lys 2000). There is no post-fledging care. The mean breeding success is 0.728 ± 0.039, and the mean survival of adults is 0.938 ± 0.002 (Rolland 2008).

Monitoring started in 1960, but all chicks were systematically ringed from 1966 to the end of this study in 2010. From mid-November to mid-December, pre-breeding adults are checked over the whole island (around 350 pairs each year during the last decade). From mid-January (just after egg laying) to mid-February, at least 3 visits are carried out every 10 days at each nest to determine the identity, sex, and breeding status (egg laid/egg hatched) of each individual. In mid-April, June, and August, all nests are monitored to check the survival of chicks. During all visits to the nests, non-breeding individuals are searched for and noted. From mid-September to mid-October, all chicks about to fledge are ringed with a stainless steel band.

### State-dependent demography: multistate model building

All 8929 individuals included in the analyses were ringed as chicks so that their exact age was known. Before their first breeding occasion, immature individuals were considered to be absent from the colony. As all individuals in this study were individually marked, and their breeding output at each breeding season carefully monitored, we built individual capture histories in a multistate format: for example 100000023045040000. This format used five observable states: ringed as chick (C) = 1 to keep track of the age of individuals in the model, seen breeding but failed at the egg-stage (FBE) = 2, seen breeding but failed at the chick stage (FBC) = 3, successful breeder (SB) = 4, seen at the colony but non-breeder (ONB) = 5. This last state (non-breeders that are observed at the colony) was included in our model because a substantial number of birds were observed as non-breeders at the colony (Table 1), probably because they were searching for a new mate. This additional information was used to estimate separately return and breeding probabilities and to improve the performance of parameter estimates (Pardo et al. 2013a). We were very confident about state assignment of this last state given the differences in breeding behavior and the numerous checks made at breeding sites.

**Table 1:** Number of observations per age and breeding state (FBE/C = failed breeder on egg/chick, SB = successful breeder, ONB = observable non-breeders). The first and last three ages were pooled for identifiability issues

State/Age	5–7	8	9	10	11	12	13	14	15	16	17	18	19	20	21	22	23	24	25	26	27	28	29	30	31	32	33	34	35	36	37	38	39	40	41	42–44	Total	%
FBE	16	58	134	188	176	119	139	113	93	113	80	95	77	76	71	49	55	50	46	41	40	45	28	26	18	19	13	13	7	5	5	7	3	2	0	2	2022	17
FBC	2	12	36	41	49	37	30	32	35	24	12	29	22	12	11	16	13	6	5	11	6	5	6	1	6	4	1	4	0	1	1	0	2	2	0	0	474	4
SB	19	158	415	547	671	624	622	593	499	508	464	416	400	334	342	289	257	241	195	187	148	137	119	108	71	73	35	45	21	17	13	14	7	6	3	2	8600	71
ONB	0	1	11	19	31	42	54	62	67	67	55	64	47	58	52	53	44	44	33	34	41	28	27	23	23	19	11	11	7	7	4	5	1	2	0	2	1049	9
All	37	229	596	795	927	822	845	800	694	712	611	604	546	480	476	407	369	341	279	273	235	215	180	158	118	115	60	73	35	30	23	26	13	12	3	6	12145	100

When an individual was not seen (coded as 0 in the capture histories), it could be because it was either not detected or emigrated permanently or temporarily, or died. In mark–recapture studies, detection probability is estimated and helps stabilizing estimations of demographic rates. Given the monitoring design described above, detection probability was high. Permanent emigration was considered negligible (Gauthier et al. 2010). The sabbatical period (i.e., corresponding to temporary emigration) could last for a year or more and was considered into the model through three unobservable states PSB, PFB, and PONB (where P stands for Post). The three “Post” states were pooled together as unobservable non-breeder (UNB). As described in Hunter and Caswell (2009), the demography of seabird species with biennial reproduction is best modeled using a multistate model that incorporates unobservable states that account for the skipped breeding behavior common in biennial breeding species.

**Figure 1:**
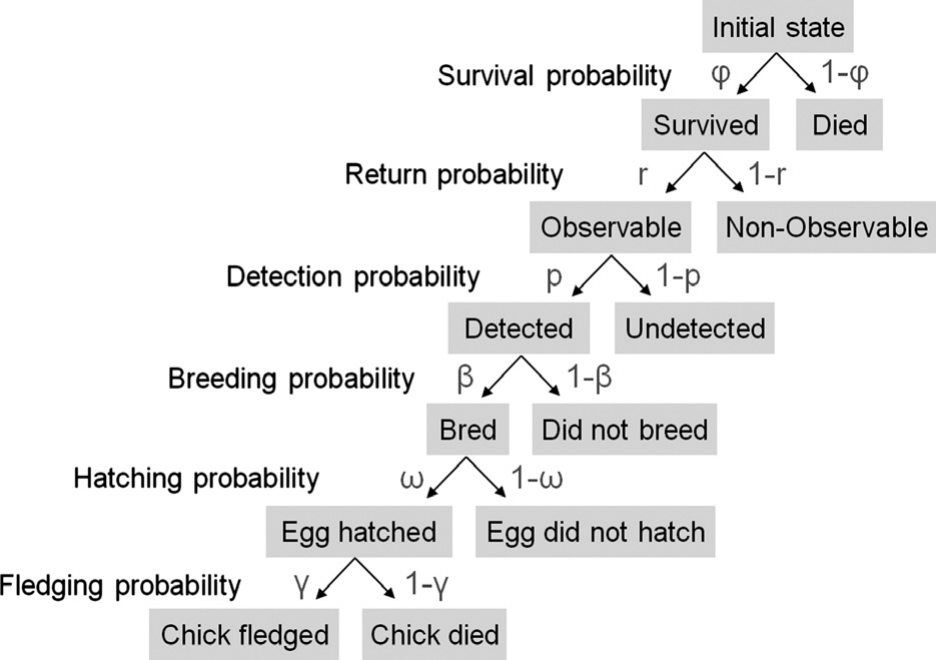
Fate of an individual in year t according to the six parameters measured: survival (*φ*), return (r), detection (p), breeding (*β*), hatching (*ω*), and fledging (*γ*) probabilities. (1-x) represents the complementary of the probability of a given parameter. To breed successfully, an individual needs to fulfill all steps by taking the left path.

To summarize the multistate model, we used had 9 different states that allowed describing individual histories in such details that we could estimate five life-history traits simultaneously (Fig. 1; Appendix S1) while taking into account imperfect detectability (p) of marked individuals at the colony: survival probability (*φ*), return probability to the breeding colony given survival (r), breeding probability given return (*β*), hatching probability given breeding (*ω*), fledging probability given hatching (*γ*) (Fig. 1; see possible transitions between states in Appendix S1; see transition matrices in Appendix S2).

States were considered to be random variables, and successive states were assumed to obey a Markov chain. Transition probabilities between states were thus modeled with a five-step procedure where survival, return, breeding, hatching, and fledging are considered as five successive steps in transition matrices. Goodness-of-fit tests were performed for multistate models (Pradel et al. 2003) using the software U-Care, version 2.3.2 (Choquet et al. 2009). We ran the test on the Jolly–Move (JMV) umbrella model with only observable states (SB, FBE, FBC, ONB), excluding the first marking of fledglings at the nest because this state was never re-observed and thus contributed no information to the test (Crespin et al. 2006).

### Effects of aging: model selection procedure

Models were run with program E-Surge, version 1.7.1 (Choquet et al. 2008). The general model was chosen to be [*φ*(.) r(f) *β*(f) *ω*(f) *γ*(f) p(f)] where each parameter was a function of the previous breeding state (f) on the four breeding parameters (r, *β*, *ω*,and *γ*) to take into account the biennial breeding tactic of this species (Hunter and Caswell 2009) and on the capture probability as we suspected differences in detection probability between birds in different states (Table 2). To avoid estimation problems, we decided to keep survival without any effect of age and previous state in the general model at the beginning (Table 2). Time effects were not considered here as models already had a very big number of parameters: 9 states, 44 age-classes, ≈ 9000 individuals, and it would have been impossible to run on any computer available to us. Aging theories predict that life-history traits should start to senesce after the age of primiparity (Hamilton 1966). Therefore, age effects were modeled starting at ten years old (the mean and modal age of first breeding). The demographic parameters of birds observed between 5 and 10 years old were estimated but are not presented here for simplicity. To achieve an efficient model selection, we chose to follow the steps of Grosbois and Tavecchia (2003). This approach permitted to solve the technical problems encountered and to compute a minimum number of alternative models. It is explained below and showed in detail in Table 2. Model selection to determine the effects of age was performed independently on each demographic trait (survival, return, breeding, hatching, fledging) and detection probability. It was started from the general model and divided in two steps (Table 2). First age effects were tested, either with age as a factor (model 1) or linear and quadratic trends of age (models 2, 3, 4 and 5 in Table 2). These models were considering either an additive effect (“+”, same slope for each previous breeding state, model 1, 2, and 3 in Table 2) or an interaction effect (“.”, different slopes for each previous breeding state model 4 and 5 in Table 2). The model with an interaction between each previous breeding state and age as a factor could not be ran because of a lack of computer memory. At that point of the model selection procedure, all demographic traits (except survival) were considering all previous breeding states separately (except UNB that were pooled together). So in a second step, while keeping the age effects selected from the first step, we examined whether some previous breeding states could be pooled together to simplify models following a rationale based on the hypothesis of differential costs of reproduction according to the previous breeding state. Several gradients were tested with respective model numbers in Table 2:

**Table 2:** Modeling the effects of age and previous breeding state on demographic traits of wandering albatrosses at Crozet Islands from 1966 to 2010. Starting from the general model, model selection was performed on each trait alternatively while keeping the general model structure in other traits. Then, the best models on each trait were combined in a composite model. For each trait, model selection was conducted in two steps. First, substeps [1,2,3,4,5] tested for age effects, either with age as a factor [1] or with linear (lin) [2,4] or quadratic (quad) [3,5] trends with age. “+” and “.” indicate that the effect of previous states was additive (same slope) or in interaction (different slopes), respectively. The interaction effect on the age-dependent model could not be tested because of the lack of computer memory. Second, substeps [6,7,8,(9)] investigated whether some previous states could be pooled together while keeping the best age structure selected in the first step according to the cost of reproduction hypotheses detailed in the method section. The pooling of states depended on the traits and the surrounding transition matrices (Appendix S2). The best model selected from the first step is showed in italics, and the final model selected after the second step is showed in bold and was kept for the composite model. “f” (from) means that the trait varied according to all previous states (FB = failed breeders on egg and chick pooled together, SB = successful breeders, B = breeders, ONB = observable non-breeders, UNB = unobservable non-breeders, NB = ONB and UNB pooled together). “_” means that the model structure is the same as the preceding step. For survival only, all previous states were pooled together (“all”) in the general model in order to obtain better parameter estimates.

Models	Survival (ϕ)	Return (r)	Breeding (*β*)	Hatching (*ω*)	Fledging (*γ*)	Detection (p)	AICc	np
General	all	f	f	f	f	f	62987.70	31
Selection on ϕ								
1	all+a	f	f	f	f	f	61956.81	76
2	f+a-lin	_	_	_	_	_	61735.55	51
3	f+a-quad	_	_	_	_	_	61890.91	52
4	***f.a-lin***	_	_	_	_	_	***61618.11***	***81***
5	f.a-quad	_	_	_	_	_	61629.17	88
6	all.a-lin	_	_	_	_	_	62379.90	45
7	B/NB.a-lin	_	_	_	_	_	61893.63	51
8	SB/FB/NB.a-lin	_	_	_	_	_	61682.87	57
9	SB/FB/ONB/UNB.a-lin	_	_	_	_	_	61713.83	63
Selection on r								
1	all	f+a	f	f	f	f	61503.17	67
2	–	f+a-lin	–	–	–	–	61492.71	36
3	–	***f+a-quad***	–	–	–	–	***61486.51***	***37***
4	–	f.a-lin	–	–	–	–	64528.55	59
5	–	f.a-quad	–	–	–	–	62357.31	66
6	–	all+a-quad	–	–	–	–	63884.39	30
7	–	B/NB+a-quad	–	–	–	–	63006.48	32
8	–	SB/FB/NB+a-quad	–	–	–	–	61735.56	33
9	–	SB/FB/ONB/UNB+a-quad	–	–	–	–	61734.18	34
Selection on *β*								
1	all	f	f+a	f	f	f	61079.57	75
2	_	_	f+a-lin	_	_	_	61073.61	44
3	_	_	f+a-quad	_	_	_	61038.40	45
4	_	_	f.a-lin	_	_	_	61006.03	74
5	_	_	*f.a-quad*	_	_	_	*60999.78*	*81*
6	_	_	all.a-quad	_	_	_	61712.86	39
7	_	_	B/NB.a-quad	_	_	_	61690.31	46
8	_	_	SB/FB/NB.a-quad	_	_	_	61708.30	53
9	_	_	**SB/FB/ONB/UNB.a-quad**	_	_	_	**60979.00**	67
Selection on *ω*								
1	all	f	f	f+a	f	f	62288.51	75
2	_	_	_	f+a-lin	_	_	62263.12	44
3	_	_	_	f+a-quad	_	_	62263.92	45
4	_	_	_	***f.a-lin***	_	_	***62260.28***	***59***
5	_	_	_	f.a-quad	_	_	62267.21	63
6	_	_	_	all.a-lin	_	_	62434.77	41
7	_	_	_	B/NB.a-lin	_	_	62284.27	47
8	_	_	_	SB/FB/NB.a-lin	_	_	62266.29	53
Selection on *γ*								
1	all	f	f	f	f+a	f	62261.53	75
2	_	_	_	_	f+a-lin	_	62263.50	45
3	_	_	_	_	*f+a-quad*	_	*61959.73*	*46*
4	_	_	_	_	f.a-lin	_	62269.73	59
5	_	_	_	_	f.a-quad	_	62261.92	63
6	_	_	_	_	all+a-quad	_	62318.21	41
7	_	_	_	_	B/NB+a-quad	_	61966.46	43
8	_	_	_	_	**SB/FB/NB+a-quad**	_	**61951.03**	**44**
Selection on p								
1	all	*f*	*f*	*f*	*f*	*f+a*	*60922.87*	*67*
2	_	_	_	_	_	f+a-lin	61244.44	36
3	_	_	_	_	_	f+a-quad	61114.67	37
4	_	_	_	_	_	f.a-lin	62995.01	47
5	_	_	_	_	_	f.a-quad	61975.43	51
6	_	_	_	_	_	all+a	62163.09	63
7	_	_	_	_	_	**B/NB+a**	**60922.66**	**65**
8	_	_	_	_	_	SB/FB/NB+a	60921.07	66
Composite	*f.a-lin*	f+a-quad	SB/FB/ONB/UNB.a-quad	*f.a-lin*	SB/FB/NB+a-quad	B/NB+a	60768.03	212

1. Breeders versus Non-Breeders at t-1 (B/NB, model 7 in Table 2)
2. Successful Breeders versus Failed Breeders versus Non-Breeders at t-1 (SB/FB/NB, model 8 in Table 2)
3. Successful Breeders versus Failed Breeders versus Observable Non-Breeders versus Unobservable Non-Breeders at t-1 (SB/FB/ONB/UNB, model 9 in Table 2).

Following the independent procedures on each of the demographic traits and detection probability, a model referred as the composite model was obtained by combining the best model structure obtained on each trait (Grosbois and Tavecchia 2003). Model selection was based on the Akaike Information Criterion corrected for small sample sizes (AICc). When ΔAICc was less than 2 between two models, the parsimony principle was applied and the model with the lowest number of parameters was selected (Burnham and Anderson 2002). As the selection subprocedures often led to local minima in the maximum likelihood estimation procedure, all models were ran with 7 random sets of initial values.

## Results

Goodness-of-fit tests (*χ*² = 1217.27; d.f. = 1274; *P* = 0.87) indicated that the general JMV model fitted the data correctly. Detection probability increased with age and was very high for individuals observed as breeders compared with non-breeders observed at the colony (Appendix S3).

### State-dependent demography

#### Survival

Contrary to our first expectation, there was not a linear link between the cost of reproduction the previous year and survival probability. The survival of unobservable non-breeders which were in sabbatical the previous year was on average across all ages higher (0.941 [mean] ± 0.082 [SE]) than the survival of observable non-breeders that returned to the breeding grounds (0.921 ± 0.027), which was higher than that of failed breeders (0.850 ± 0.065). However, birds that succeeded to fledge a chick had the highest survival probability (0.943 ± 0.024).

#### Global pattern: probability of breeding successfully

**Figure 2:**
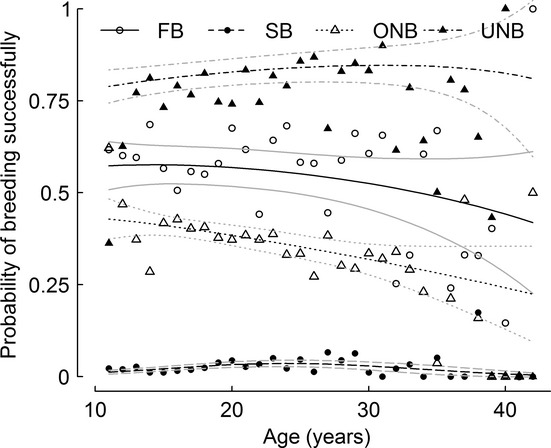
Global patterns: age-dependent variations in the synthetic reproductive trait probability of breeding successfully calculated as the product of return, breeding, hatching, and fledging probabilities according to four breeding states the previous year. Global patterns: age-dependent variations in the synthetic reproductive trait probability of breeding successfully calculated as the product of return, breeding, hatching, and fledging probabilities according to four breeding states the previous year. Black lines are model estimates, gray lines stand for the 95% confidence intervals and symbols for raw data. Plain line and empty circles for failed breeders (FB), dashed line and filled circles for successful breeders (SB), dotted line and empty triangles for observable non-breeders (ONB) and dash-dot line and filled triangles for unobservable non-breeders (UNB).

To describe how the different reproductive traits varied according to the previous breeding state, we calculated the probability of breeding successfully as the product of return, breeding, hatching, and fledging probabilities (Fig. 2). This integrative measure is often represented in studies at the population level when no detailed information on breeding states is available. The probability of breeding successfully varied according to the previous breeding state with a clear gradient showing highest mean performances in birds that were absent from the colonies the previous year compared with the ones that were present, even if not breeding. Indeed birds that took a sabbatical year (unobservable non-breeders) had a probability of breeding successfully of 0.829 ± 0.032 the next year. Birds that bred the year before, thus breeding two consecutive years, had much lower chances of breeding successfully, respectively, 0.528 ± 0.041 and 0.023 ± 0.004 for birds that failed or succeeded the previous year. Birds that were observable at colonies without breeding the previous year had a surprisingly low probability of breeding successfully of 0.337 ± 0.027.

### Effects of aging

#### Survival

**Figure 3:**
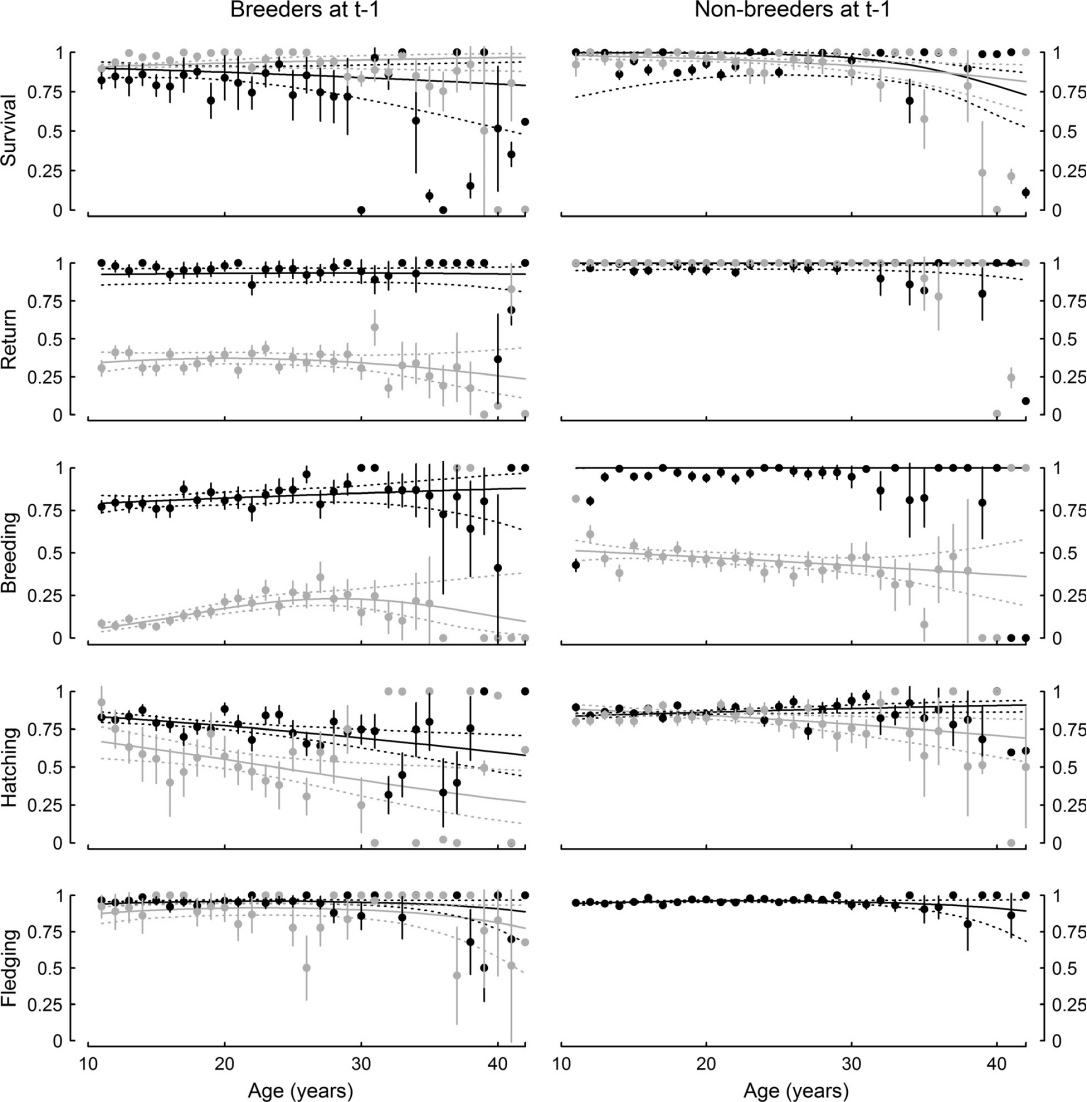
Detailed patterns: age-dependent variations in survival, return, breeding, hatching, and fledging probabilities. Breeders at t-1 are represented birds that failed (black line, dark gray 95% CI) and birds that succeeded (gray line, light gray 95% CI). Non-breeders at t-1 are represented birds that did not attend the colony (black line, dark gray 95% CI) and birds that came back to the colony (gray line, light gray 95% CI). Dots represent the data obtained with a full age-dependent model (±SE). More details on the best model selected on each trait can be found in Table 1. Ages from 41 to 43 were pooled together to avoid rank deficiency. For simplicity, only ages >10 are represented.

Model selection suggested that survival probability was different for all previous breeding states, but some estimates in late age and for breeders that were successful the year before were imprecise (data point limited at one or zero on Figure 3) due to small sample sizes at old ages. The survival of unobservable and observable non-breeders was close to 1 at the youngest breeding ages until 25 years old. Then survival started to decrease substantially to, respectively, 0.730 ± 0.104 and 0.815 ± 0.098 at the oldest ages. Concerning breeders the previous year, survival probability of both failed and successful birds was similar at the youngest ages, respectively, 0.899 ± 0.032 and 0.910 ± 0.009. Then the survival of individuals that failed the year before decreased to 0.799 ± 0. 159, whereas it increased for successful breeders to 0.968 ± 0.045 (Fig. 3).

#### Global pattern: probability of breeding successfully

When considering aging effects on the probability of breeding successfully, results suggest that previous observable non-breeders and failed breeders showed the steepest and most steady decrease in this parameter at late age, whereas the probability of breeding successfully of unobservable non-breeders increased at middle ages and decreased only slightly at the oldest ages (Fig. 2). The pattern in successful breeders the previous year was intermediate: The probability of breeding successfully increased from 0.012 ± 0.003 in young inexperienced breeders at 11, to 0.036 ± 0.004 in middle-aged individuals. However, at the oldest ages, this probability decreased to 0.004 ± 0.003 and very few birds fledged a chick two years in a row. To understand the contribution of each reproductive trait to these age-related patterns in the probability of breeding successfully, we investigated the detailed patterns for each reproductive trait.

#### Detailed patterns in reproductive traits

Return probability was ≈ 1 in birds that did not breed the year before irrespective of their age. In breeders, it was much higher for individuals that failed the year before than for the ones that succeeded, respectively, 0.931 ± 0.019 and 0.344 ± 0.033 at age 11. Furthermore, at the oldest ages, whereas return probability remained constant for failed breeders the previous year at 0.927 ± 0.060, it decreased substantially in successful breeders to 0.236 ± 0.065 at the oldest ages (Fig. 3).

The patterns in breeding probability were more contrasted. Breeding probability of birds that were in sabbatical the year before was ≈ 1 irrespective of their age. However, breeding probability of birds that attended the colony without breeding the year before was 0.548 ± 0.016 at 11 years old and decreased strongly with age to 0.314 ± 0.033 at age 42. In breeders, as expected, breeding probability was higher in birds that failed in their breeding attempt the previous year than in birds that succeeded. Surprisingly, breeding probabilities of failed breeders the previous year increased with age, from 0.838 ± 0.026 at 11 to 0.969 ± 0.126 at the oldest ages (Fig. 3). In successful breeders, the breeding probability was selected to be bell-shaped, showing an increase from young to middle-aged birds to reach the maximal value of 0.232 ± 0.021 at 28 years old. Then at the oldest ages, the breeding probability of birds that were successful the year before potentially decreased, but the analysis suffered from a lack of data and it is possible that the curve stabilizes or even increases as for failed breeders, given the very large 95% confidence intervals.

Except for unobservable non-breeders, hatching probability decreased with age whatever the previous state of the birds. At 11 years old, it was 0.850 ± 0.016 for both observed non-breeders and failed breeders the previous year, whereas it was of 0.670 ± 0.051 for successful breeders in the previous year. For the oldest individuals, the effect of the previous breeding state became more important with a hatching success probability of 0.744 ± 0.059, 0.576 ± 0.074, and 0.266 ± 0.089 in observable non-breeders, failed breeders, and successful breeders the previous year, respectively (Fig. 3).

Finally, fledging success probability was similar between individuals that did not breed or failed the previous year (0.961 ± 0.004 and 0.959 ± 0.006 at age 25, respectively), and slightly lower for successful breeders (0.911 ± 0.021 at age 25). Fledging success decreased slightly with age starting at 30 years old whatever the previous breeding state (Fig. 3).

## Discussion

Based on a multistate and multitrait approach, this study allowed disentangling the complex linkages between breeding state and aging. We showed that the influence of age on several demographic traits varied according to the previous breeding state. At the population level, following a strict biennial breeding frequency led to reduced age effects on reproductive performance even at ages older than 40. Reproductive senescence was more marked in birds attempting to breed annually and in a category of birds often neglected: observable non-breeders, that is, birds visiting the colony without breeding, suggesting intricate underlying mechanisms. Interestingly, the probability of engaging in a reproductive attempt the year following a reproductive failure increased with age. We found actuarial senescence but, contrary to our predictions, it affected more strongly non-breeders and failed breeders the year before, whereas the survival of successful breeders remained very high even after 40 years old.

### State-dependent demographic performances

Among Procellariiformes there is a continuum of breeding frequencies, ranging from strictly biennial to typical annual species (Jouventin and Weimerskirch 1988; Chastel 1995; Jouventin and Dobson 2002). Biennial breeding is unusual among animal species and is generally observed in long-lived species with a particularly long-lasting breeding season. Wandering albatrosses stand at the extreme position of this gradient, due to their one year long breeding season. Nevertheless, as recently demonstrated by Barbraud and Weimerskirch (2011), we found that a non-negligible proportion of birds attempted to breed annually after a successful breeding attempt. Such individuals thus have only a few weeks between the end of the previous breeding cycle and the coming one to restore their body condition and molt. This short period is not long enough for molting that takes place over months, especially since the duration of the interbreeding season affects directly the extent of molt in wandering albatrosses (Weimerskirch 1991). Molt is energetically costly and essential as worn flight feathers have been demonstrated to negatively influence current breeding success and future breeding probability (Weimerskirch 1991; Rohwer et al. 2011).

Wandering albatrosses were good biological models for investigating the effect of previous breeding state on life-history traits. Our first prediction was that the breeding performances and survival should increase gradually as the potential costs of reproduction were lower the previous year. This was clearly demonstrated for the probability of breeding successfully which was lowest for birds that were successful breeders the previous year and highest for those that were not observed on land the previous year. However, we would have expected non-breeders visiting the colonies to have a lower probability of breeding successfully than failed breeders the previous year (early and late failures pooled), which was not the case. The particular case of observable non-breeders is discussed below. The detailed patterns in reproductive parameters allowed us to demonstrate that the differences between previous breeding states varied according to the life-history trait considered.

### Effects of aging

Overall, there were only relatively small effects of age on the reproductive performance of birds that skipped one or more years before their breeding attempt. However, some individuals attempted to increase their breeding frequency by skipping a sabbatical year and breeding immediately again after having fledged a chick. These individuals showed the steepest decreases in all their reproductive traits: probabilities of return, breeding, hatching, and fledging, supporting our second prediction. Decreases in performance were linear from the age of primiparity except for the fledging probability where the onset of senescence was earlier in birds that had a successful breeding attempt the previous year. For birds that failed early or late during the previous breeding attempt, the changes in reproductive performance with age were intermediate. We can question about the existence of two breeding tactics in this population. Of the 838 individuals that attempted to breed at least once after a successful breeding on average 20% (± 11.6%) of their entire recorded breeding attempts were annual.

It is interesting to insert the rates of senescence identified in this article into a gradient of short- to long-lived birds and mammals species (e.g., in Bouwhuis et al. 2012). As this study had missing points for long-lived bird species, we have added our results to their graphs; this can be seen in Pardo et al. (in press). We notice that as expected, wandering albatrosses present very low fitness cost of senescence compared with mammals of equivalent survival, in females in particular.

Yet one surprising pattern arose for the breeding probability of failed breeders the previous year. Instead of decreasing as for the other category of breeders and for observable non-breeders, it increased significantly with age. Such an increase in breeding frequency in late age was detected in several long-lived species: blue-footed booby (*Sula nebouxii*; Velando et al. 2006), red deer (*Cervus elaphus*; Clutton-Brock 1984), moose (McElligott et al. 2002), and reindeer (*Rangifer tarandus*; Weladji et al. 2010). In ungulates, females seemed to increase calf body condition to compensate reproductive senescence in other traits as litter size and parental care. In our case, the increase in breeding frequency could represent such compensation to the highlighted senescence in hatching and fledging success. Although previously failed breeders did not present the strongest senescence, we believe it is possible that previously successful breeders also exhibit an increase in their breeding probability that may have been masked due to limited sample size on successful breeders that attempt to breed again when aged more than 35 years old. As they age, wandering albatrosses may benefit from increasing their breeding probability as a form of terminal investment if an individual's state in the broad sense is depleted by the accumulation of internal damage linked to senescence effects (McNamara et al. 2009). Indeed the mean age of successful breeders breeding during consecutive years (20.3 ± 0.4, 261 observations) was significantly higher than the mean age of successful breeders after one or more sabbatical years (17.9 ± 0.1, 6885 observations).

### Trade-offs between reproduction and survival

If average reproductive performance and senescence patterns in hatching and fledging probabilities are strongest in birds that attempt to breed two years in a row, one can thus wonder the potential underlying reasons why a substantial number of individuals engage in this strategy. The decision of breeding in general depends on body condition (Weimerskirch 1992), and it might be reasonable to assume that the decision to breed following a successful breeding attempt depends also on body condition. Body condition apart from age can be associated with environmental covariates as proxies of prey availability as demonstrated in blue petrels (*Halobaena caerulea*; Chastel et al. 1995) and red-footed boobies (*Sula sula;* Cubaynes et al. 2011), or hormonal levels as in female Galápagos marine iguanas (*Amblyrhynchus cristatus*; Vitousek et al. 2010) and snow petrels (*Pagodroma nivea*; Goutte et al. 2011). One hypothesis could be that in years of good environmental conditions, the number of wandering albatrosses breeding two years in a row increases. Also, the response of individuals to the environment may vary according to their age as we demonstrated in another albatross species (Pardo et al. 2013b). This remains to be tested in wandering albatrosses but until now few effects of environmental variability have been demonstrated in this species compared with other albatrosses (Rolland et al. 2010).

Another explanation can stand in the shape of the relationship between survival and age. Indeed we expected to find the strongest senescence rates and earliest onset of actuarial senescence in birds that attempted to breed two years in a row, but the opposite pattern arose. Both previously observable and unobservable non-breeders had their survival probability lower than 0.7 which is low for such a long-lived species. On the other side, the survival probability of previously successful birds reached very high values and presents no senescence, whereas the one of previously failed breeders showed an intermediate rate of senescence. This is not in accordance with what other studies found when investigating the trade-offs between reproduction and survival according to the previous breeding state. Indeed in Asian elephants (*Elephas maximus*), North American ground squirrels (*Tamiasciurus hudsonicus*) and Soay sheep (*Ovis aries*), the survival probability of the oldest individuals was lower for breeders than non-breeders (Tavecchia et al. 2005; Descamps et al. 2009; Robinson et al. 2012). Our results thus suggest that another process than the costs of reproduction may shape the age-related life-history traits of wandering albatrosses according to their previous breeding state. The lowest senescence rates and potentially increased breeding probabilities with age of previously successful and failed breeders may be influenced by a selective disappearance process (for theory see Vaupel and Yashin 1985). Indeed, it is possible that lower-quality individuals disappear from the population at an earlier age, thus increasing the proportion of high-survival individuals. The existence of higher quality individuals leading to heterogeneities in survival probabilities has been demonstrated in an array of other bird species as black-legged kittiwakes (*Rissa tridactyla*, Cam et al. 2002), black-headed gull (*Chroicocephalus ridibundus*, Peron et al. 2010), oystercatcher (*Haematopus ostralegus,*Van de Pol and Verhulst 2006). However, as we decomposed reproduction into four different probabilities according to the previous breeding state, we can also see here that the high-survival individuals remaining in late life probably may manage to restore their body condition faster between two breeding episodes. This means that they could better tolerate breeding two years in a row even at old ages despite the absence of molting and reduced foraging issues discussed above. Such birds even appeared to increase their breeding probability after a failed breeding event and potentially a successful breeding event whereas others, even if strictly biennial breeders, may be more likely to die. Models incorporating individual heterogeneity should thus be tested in future studies to refine these results of state-dependent aging as they may reveal masked patterns of senescence. Indeed, Peron et al. (2010) have shown that senescence is more detectable when individual heterogeneity is accounted for. So the senescence patterns found in this article could be underestimated. However, it may help disentangling potential classes of individuals that increase breeding probability, for instance, whereas others tend to limit their costs of reproduction.

### The specific case of observable non-breeders

Observable non-breeders represented approximately one-tenth of all non-breeders (Bonnevie et al. 2012). Whereas most demographic studies ignored this subset of the populations, we chose to use this information in our multistate model because it allowed decomposing totally the reproduction process. Indeed, when some birds are not detected, they may either be absent from the breeding grounds or may have returned but being overlooked. Therefore, by incorporating these observable non-breeders, we could differentiate between detection and return probabilities. This information not only allowed us to improve parameter estimates of breeding and detection probabilities (Pardo et al. 2013a), but also to focus on aging for birds in this state in this wandering albatross population. Observable non-breeders had different responses than expected in particular on breeding and hatching traits. We expected them to have similar traits and trait variation according to age than unobservable non-breeders but they had lower average reproductive performance and stronger senescence rate. We know from a sister study investigating the sex-related differences in aging in this species (Pardo et al. in press) that almost 75% of ONB birds were males and they seemed to be entering a succession of non-breeding events during which they continue to return at breeding grounds while their breeding probability declines progressively as they age. These males might be searching for mates after the loss of their partner and do not manage to find one given the skewed sex ratio of the population due to female-biased mortality in longline fisheries (Weimerskirch and Jouventin 1987; Weimerskirch et al. 2005).

## Conclusions

This study allowed identifying both actuarial and reproductive senescence and results highlighted heterogeneity in individual breeding tactics that became particularly apparent when age was taken into account. Birds that attempted to breed immediately following a previous breeding attempt exhibited stronger senescence on breeding parameters, whereas birds that followed a biennial breeding tactic showed almost no change at old ages. A surprising increase in breeding frequency was detected for individuals attempting to breed two years in a row and may represent a form of terminal investment to possibly compensate for low breeding success at the oldest ages. The patterns observed on survival probability were contrary to our expectations which suggested an influence of individual quality rather than trade-offs between reproduction and survival at late ages. This study demonstrated the importance of taking into account the information brought by all different breeding status as failed and successful breeders but also observable and unobservable non-breeders. More generally, these results revealed important mechanisms that increase our knowledge of the evolutionary ecology of senescence and highlight the benefits of multitrait approaches.

## Supporting Information

**Appendix S1**. Life cycle graph representing transitions between the nine states. **Appendix S2.** Transition matrices from GEPAT in E-SURGE. **Appendix S3.** Detection probabilities according to age for the birds that are breeders and non-breeders at the colony at a given year.
