# Supplementary material for: What shall I do now? State-dependent variations of life-history traits with aging in Wandering Albatrosses
Source: Ecol Evol. 2014 Jan 23;4(4):474–87. doi: 10.1002/ece3.882 (PMC3936393; doi:10.1002/ece3.882)
Supplement: Supplementary file 1 — Appendix S1. Life cycle graph representing transitions between the nine states. Appendix S2. Transition matrices from GEPAT in E-SURGE. Appendix S3. Detection probabilities according to age for the birds that are breeders and non-breeders at the colony at a given year. [file ece30004-0474-sd1.doc]

Appendix S1: Life cycle graph representing transitions between the nine states. The chick stage (C) including immaturity is represented in light grey. Chicks can only pass into breeding states at the minimum age of 5. Observables states are represented in white. They include the three breeding states: Successful Breeder (SB), Failed Breeder on Egg (FBE) and Failed Breeder on Chick (FBC) and the Observable Non-Breeding state (ONB). The transitions between observable states are represented by bold double arrows. The transitions between an observable state and its corresponding unobservable state are represented with solid double arrows. The three unobservable states are represented in dark grey with Post-Successful Breeders (PSB), Post-Observable Non-Breeders (PONB) and Post-Failed Breeders (PFB). The transitions from unobservable states to observable states are represented in dashed arrows. The formulas on top of each state correspond to the probabilities of being in each state at time t, where φ, r, β, ω, γ, ρ represent respectively the survival, return, breeding, hatching, fledging and detection probabilities and (1-x) is the complementary.

Appendix S2: Transition matrices from GEPAT in E SURGE. At the Initial state, all individuals are in the Chick stage since only birds ringed as chicks were considered. Then five transitions are considered from breeding states at time t (rows of the matrices) to breeding states at time t+1 (columns of the matrices): survival (s), return (r), breeding (β), hatching (ω) and fledging (γ). Detection probabilities of Chicks were fixed to 1. F = Failed, S=Successful, B=Breeder, E=Egg, C=Chick, O=Observable, U=Unobservable, N/n=Non, P=Post, †=dead. “_” represents the absence of possible transition and “*” is the complementary. 1B stands for first breeders, r/nr for return or non-return, β/n β for breeding or not-breeding, “_ ω” for incubating.

Appendix S3: Detection probabilities according to age for the birds that are breeders and non-breeders at the colony at a given year. Dots are mean values and the uncertainty is represented by standard deviations.
